# Image Tracing of Inflammatory Intestinal Organoids via Computational Clearing

**DOI:** 10.3390/nano16100629

**Published:** 2026-05-19

**Authors:** Dong-Gyu Jeon, Min-Young Han, Hana Lee, Hanguk Hwang, Ji-Min Lee, Eun Soo Kim, Gang Ho Lee, Yongmin Chang, Mi-Young Son, Mae-Ja Park, Sung-Wook Nam

**Affiliations:** 1 Department of Molecular Medicine, School of Medicine, Kyungpook National University, Daegu 41405, Republic of Korea; 2 Cell & Matrix Research Institute, Kyungpook National University, Daegu 41944, Republic of Korea; 3 Department of Biomedical Science, Kyungpook National University, Daegu 41944, Republic of Korea; 4 Stem Cell Convergence Research Center, Korea Research Institute of Bioscience and Biotechnology (KRIBB), Daejeon 34141, Republic of Korea; 5 Division of Gastroenterology, Department of Internal Medicine, School of Medicine, Kyungpook National University, Daegu 41944, Republic of Korea; 6 Department of Chemistry, College of Natural Sciences, Kyungpook National University, Daegu 41566, Republic of Korea; 7 Department of Anatomy, School of Medicine, Kyungpook National University, Daegu 41944, Republic of Korea

**Keywords:** intestinal organoids, image tracing, dextran sulfate sodium, inflammation, computational clearing, image segmentation, image morphometrics

## Abstract

Computational clearing (CC) enhances widefield (WF) fluorescence microscopy by suppressing out-of-focus haze and autofluorescence, yielding semi-confocal quality images suitable for segmentation and image-based phenotyping. Here, we propose an “image tracing” workflow for inflammatory mouse intestinal organoids (mIOs) using paired CC and WF images to generate a differential signal (CC − WF). mIOs were derived from intestinal crypts of Lgr5-EGFP stem cell reporter mice and expanded under epidermal growth factor, Noggin, and R-spondin (ENR) conditions. Inflammation was induced by dextran sulfate sodium (DSS) treatment. CC processing enhanced phalloidin-stained apical F-actin and improved EGFP signals by reducing background noise, enabling robust segmentation and quantitative extraction of image morphometrics including area, circularity, and perimeter. CC-WF vectors derived from three-dimensional area–perimeter–circularity plots sensitively captured DSS-induced epithelial disruption analogous to a leaky-epithelium phenotype. Transcriptomic analysis by RNA-seq of DSS-treated mIOs revealed upregulation of inflammatory pathways including TNF-α signaling via NF-κB and IL-6/JAK/STAT3, aligning with microscopy findings. In a proof-of-concept demonstration using phalloidin-stained fluorescence images, ROC analysis of the CC-WF workflow achieved an AUC = 0.95 with 87.5% sensitivity and 92.9% specificity in distinguishing intact from injured mIOs.

## 1. Introduction

Imaging technologies have revolutionized biomedical research by enabling the visualization of gene and protein expression at the cellular level [1,2,3,4,5,6]. Recently, three-dimensional organoid structures have emerged as promising models due to their ability to mimic the physiology of various organs including the intestine, brain, lung, and kidney [7]. A wide range of imaging tools has been developed, including super-resolution microscopy [8], light-sheet microscopy [9], and microchip-assisted microscopy [10,11,12,13,14,15]. In parallel with advances in microscopy hardware, novel sample preparation methods enhance image quality, such as tissue clearing [16] and expansion microscopy [17].

Despite significant advances in imaging technology, fluorescence microscopy still faces limitations, particularly signal blurring due to sample thickness and strong background noise [18]. These artifacts can be corrected using image processing algorithms such as deconvolution microscopy that reduce background noise while enhancing the signal-to-noise ratio [19,20,21,22]. Although these processes substantially improve image quality, few approaches actively utilize this enhancement for practical diagnostic applications, as image blurring is typically regarded simply as a technical artifact to be removed.

We propose a method to leverage image processing effects for medical diagnostic applications. Computational clearing (CC) is a recently developed algorithm that performs image correction by removing background noise [23,24]. Notably, during this process, specific fluorescence signals regarded as true signals are further enhanced, while broad signals from non-specific antibody binding are minimized. Our hypothesis is that this CC method can enable discrimination between intact and injured states of organoids in fluorescence microscopy images, analogous to pathological scoring. To test this, we utilized the difference between CC and original widefield (WF) images, employing image segmentation to extract image morphometrics including object area, perimeter, and circularity. Like lineage tracing to track stem cell-induced differentiation [25,26,27,28], we termed this approach “image tracing” by tracking the image processing procedures to extract diagnostic meaning.

In this report, we introduce an inflammatory model of mouse intestinal organoids (mIOs) through the treatment of dextran sulfate sodium (DSS) to examine the image tracing workflow. DSS is a well-established agent for inducing intestinal inflammation models both in vivo and in vitro [29]. mIO cultures were established by isolating crypts from EGFP-tagged *Lgr5* reporter mice (*Lgr5-EGFP-IRES-creERT2*), in which *Lgr5* marks intestinal adult stem cells [30,31]. Long-term culturing of mIOs was established through more than 10 passages. The growth of mIOs was confirmed by the identification of Lgr5+ stem cells flanked by Paneth cells. The cultured mIOs were treated with DSS to induce an in vitro inflammation model. Through CC image processing, we obtained high-quality images and quantified the area, perimeter, and circularity of each organoid via image segmentation. The key step is to extract the difference between CC and WF images (CC − WF) to distinguish control and inflammatory organoids. This image tracing approach enabled us to achieve diagnostic performance with an AUC of approximately 0.95 for distinguishing between control and DSS-treated organoids.

## 2. Materials and Methods

### 2.1. Isolation of Crypts from Mouse Intestine

*Lgr5-EGFP-IRES-creERT2* mice (Jackson Laboratory, Bar Harbor, ME, USA) were sacrificed to isolate intestinal crypts. The jejunum was excised, rinsed with cold Dulbecco’s phosphate-buffered saline (DPBS), and placed in a 10 cm Petri dish. The intestinal wall was carefully opened longitudinally, and the luminal surface was scraped multiple times using a thin coverslip to remove debris. Subsequently, the intestine was cut into 3–5 mm fragments and transferred to cold DPBS in a 50 mL conical tube. The fragments were vigorously shaken and rinsed repeatedly with cold DPBS to ensure thorough cleaning.

Intestinal fragments were then transferred to 10 mL gentle cell dissociation reagent (GCDR, STEMCELL Technologies, Vancouver, BC, Canada) and incubated at room temperature for 15 min while being gently rocked on the shaker. The GCDR solution containing treated intestinal fragments was passed through a 70 μm cell strainer into 30 mL cold DPBS to isolate intestinal crypts in the filtrate.

### 2.2. Formation of Matrigel Domes for Culturing Mouse Intestinal Organoids

The filtered crypt solution was centrifuged at 300× *g* for 5 min. The pellet was resuspended in 10 mL Dulbecco’s modified Eagle’s medium (DMEM)/nutrient mixture F-12 (STEMCELL Technologies). The suspension was centrifuged at 600 × *g* for 5 min, and the pellet was resuspended in a 1:1 mixture of IntestiCult Organoid Growth Medium (OGM), mouse basal medium (STEMCELL Technologies) and Matrigel (Corning, NY, USA, Cat. No. 354230). A 50 μL dome was formed in each well of a 24-well microplate. After incubation at 37 °C for 30 min to allow Matrigel polymerization, 750 μL OGM was added to each well.

### 2.3. Drug Preparation

Dextran sulfate sodium (DSS, Cayman Chemical, Ann Arbor, MI, USA) was dissolved in distilled water at a concentration of 1% (weight per volume) (0.5 g DSS in 50 mL water) and stored at −80 °C. For experimental treatments, 60 μL of the concentrated DSS stock solution was diluted in 120 μL OGM to achieve a 1:3 dilution, which was further serially diluted to obtain the desired concentrations.

### 2.4. Immunocytochemistry

For fluorescence microscopy observation, mouse intestinal organoids (mIOs) were cultured in microplates. After culture, mIOs were fixed in 4% paraformaldehyde (Sigma-Aldrich, St. Louis, MO, USA) in PBS for 15 min at room temperature. Following permeabilization with 0.1% Triton X-100 (Sigma-Aldrich, St. Louis, MO, USA) for 10 min, cells were blocked with 5% bovine serum albumin (BSA) solution for 1 h at room temperature. mIOs were stained with Phalloidin-AlexaFluor 488 (Invitrogen, Carlsbad, CA, USA) and DAPI (Thermo Fisher Scientific, Waltham, MA, USA). Stained organoids were visualized using an inverted fluorescence microscope equipped with computational clearing technology (THUNDER Imager DMi8, Leica Microsystems, Wetzlar, Germany).

### 2.5. Image Segmentation

For AIVIA software analysis, we loaded TIFF images and performed manual sketches on both the target object and the background (Supplementary Figure S10). In our case, organoid features in microscopy images and the background were separately sketched in the localized area. Based on the user’s sketch, AIVIA software automatically expands the sketch to the entire image region and performs image segmentation procedures based on an embedded AI algorithm.

### 2.6. Live–Dead Cell Assay

Grown mIOs were treated with DSS at various concentrations for 3 days. A live–dead cell assay was performed using the ReadyProbes Cell Viability Imaging Kit (Invitrogen) according to the manufacturer’s instructions. Fluorescence spectroscopy was measured using a SpectraMax iD3 microplate reader (Molecular Devices, San Jose, CA, USA). Fluorescence images were obtained using an EVOS M7000 imaging system (Thermo Fisher Scientific, Waltham, MA, USA). Fluorescence spectra and fluorescence images were correlated to validate viability measurements.

### 2.7. Cell Viability Test

Viability testing was performed on mIOs treated with DSS at concentrations ranging from 10,000 to 0.5 μg/mL for 3 days. Cell viability was assessed using the CellTiter-Glo 3D Cell Viability Assay (Promega, Madison, WI, USA). Luminescence was measured using a SpectraMax iD3 microplate reader (Molecular Devices).

### 2.8. Total RNA Extraction

For gene expression analysis, DSS-treated mIOs were harvested as follows. After washing Matrigel domes with DPBS, TrypLE Express (Gibco, Thermo Fisher Scientific, Waltham, MA, USA) was added while disrupting the dome with gentle pipetting. Samples were incubated at 37 °C for 15 min for enzymatic digestion. The cell suspension was transferred to a 15 mL conical tube and centrifuged at 600× *g* for 5 min. Cell pellets were lysed in 350 μL lysis buffer from the RNeasy Mini Kit (Qiagen, Hilden, Germany), and total RNA was extracted according to the manufacturer’s instructions. Total RNA concentration and purity were measured using a NanoDrop spectrophotometer (Thermo Fisher Scientific).

### 2.9. cDNA Synthesis and Reverse Transcription Polymerase Chain Reaction

cDNA was synthesized from total RNA using the RevertAid First Strand cDNA Synthesis Kit (Thermo Fisher Scientific) according to the manufacturer’s instructions, yielding 100–150 μL cDNA. Primers (Bioneer, Daejeon, Republic of Korea) for intestinal cell markers, including stem cells (*Lgr5*), Paneth cells (*Lyz1*), enterocytes (*Vil1*), goblet cells (*Muc2*), and enteroendocrine cells (*Chga*), were designed and synthesized (primer sequences are listed in Supplementary Table S1). Quantitative polymerase chain reaction (qPCR) was performed by mixing cDNA and primers with Premix Ex Taq Hot Start master mix (Takara Bio, Kusatsu, Japan) using a CFX Opus 96 Real-Time PCR System (Bio-Rad, Hercules, CA, USA).

### 2.10. Statistical Analysis

Data from cell viability tests and live–dead assays were analyzed and plotted using GraphPad Prism version 11.0 (GraphPad Software, San Diego, CA, USA). Data are presented as mean ± standard deviation (SD). Statistical significance was analyzed by one-way analysis of variance (ANOVA) with post hoc testing. The *p*-values less than 0.05 were considered statistically significant (* *p* < 0.05, ** *p* < 0.01, *** *p* < 0.001, **** *p* < 0.0001). Image profiles from live–dead assays were analyzed using ImageJ version 1.54 (National Institutes of Health, Bethesda, MD, USA) and plotted using OriginPro version 10.1 (OriginLab, Northampton, MA, USA).

## 3. Results

### 3.1. Hypothesis for Image Tracing (CC-WF) of Injured Organoids

**Figure 1:**
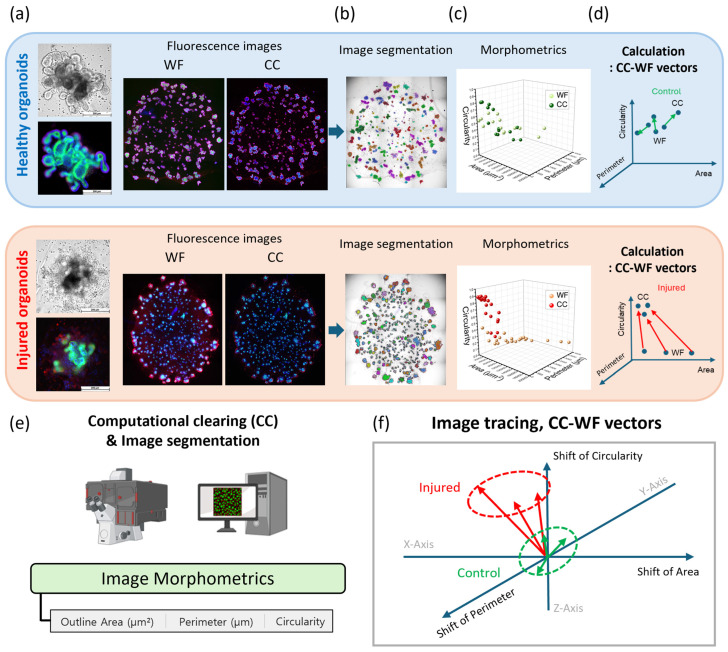
Image tracing workflow for healthy and injured organoids. (**a**) Widefield (WF) fluorescence images were processed by computational clearing (CC). (**b**) The CC images were segmented for the quantification of organoid shapes. (**c**) Morphometrics such as area, circularity, and perimeter were extracted. (**d**) From 3D scatter plots of area, perimeter, and circularity, CC-WF vectors were calculated. (**e**) Schematics of CC and image segmentation procedures to extract image morphometrics. (**f**) Principle of image tracing using CC-WF vectors to distinguish injured organoids from control organoids.

The image tracing workflow was designed to quantify the integrity state of organoids. This methodology is based on computational clearing (CC) processing of widefield (WF) fluorescence images using the THUNDER microscope (Leica Microsystems), which implements a recently developed image processing algorithm [18,23,24] (Figure 1a). Bright-field and fluorescence images of mIOs were acquired. From WF fluorescence images stained with phalloidin, DAPI, and ZO-1, we selected the phalloidin channel due to its clear definition of the apical side of the organoid lumen [32,33].

The CC algorithm is conceptually similar to conventional deconvolution algorithms, in which an estimated point spread function (PSF) is deconvolved from the WF microscopy image to extract the original specimen signal. One distinguishing feature of CC is that the applied PSF is differentiated between the in-focus and out-of-focus regions, referred to as “length-scale discrimination” of the PSF [24]. The WF image can be decomposed into in-focus and out-of-focus contributions, each described by its respective PSF. Because the out-of-focus contribution typically appears as a broad and slowly varying background, much larger in spatial scale than the fine features of the specimen, the CC algorithm iteratively identifies and isolates this large-scale background, while leaving the smaller in-focus structure intact [24]. Therefore, it is assumed that only background noise is removed, while the in-focus signal, edges, and relative intensities of specimen features are preserved. In this report, the instant CC mode was applied, which performs single-image real-time background removal using a feature scale parameter matched to the organoid diameter (~400–500 µm) in the LAS X software version 3.7.5.24914 (Leica Microsystems) (Supplementary Figure S1).

Based on the CC-processed images, image segmentation was performed using AIVIA software version 15.0 (Leica Microsystems, Wetzlar, Germany), an AI-powered image analysis platform that employs a Cellpose-based deep learning segmentation algorithm (Figure 1b) [34]. Unlike conventional threshold-based tools such as OrganoSeg or ImageJ [35,36], AIVIA utilizes pre-trained models for object detection, enabling robust segmentation without manually defined thresholding parameters. At the same time, the image segmentation produces image morphometrics including organoid area (μm^2^), perimeter (μm), and circularity (Figure 1c).

Our hypothesis proposes that organoid integrity is reflected not only in the CC image itself, but also in the differential signal between CC and WF images (Figure 1d). We explored this possibility by obtaining CC-WF vectors for image-segmented objects from paired CC and WF images. WF images were processed by CC for both healthy and injured organoids. The resulting images (WF healthy, CC healthy, WF injured, and CC injured) were segmented. An important part of our suggestion is to extract morphological metrics, including area, perimeter, and circularity, from the segmented CC and WF images (Figure 1e). The area, perimeter, and circularity values were plotted in 3D scatter graphs, and vectors were calculated from each WF point to its corresponding CC point. This vector, representing the shift from WF to CC, is defined as the CC-WF vector (Figure 1f).

Recently, statistical analysis of organoid morphology has been widely examined alongside AI algorithms [37]. In our study, we employed the CC algorithm to validate our hypothesis that CC-WF vectors can distinguish between intact and inflammatory mIOs. For intact organoids, we hypothesize that the CC-WF vector exhibits minimal shift because the WF fluorescence signal originates from highly specific staining of target proteins through authentic antibody–antigen binding, and the resulting CC images faithfully represent true signals. In contrast, for injured organoids where target proteins are damaged, the WF fluorescence signal arises from non-specific antibody binding, and CC processing generates exaggerated or distorted images. Consequently, the CC-WF vector of injured organoids exhibits a substantial shift, which we hypothesize serves as the key diagnostic feature in the CC-WF image tracing workflow.

### 3.2. Long-Term Culture of Lgr5-EGFP Intestinal Organoids

**Figure 2:**
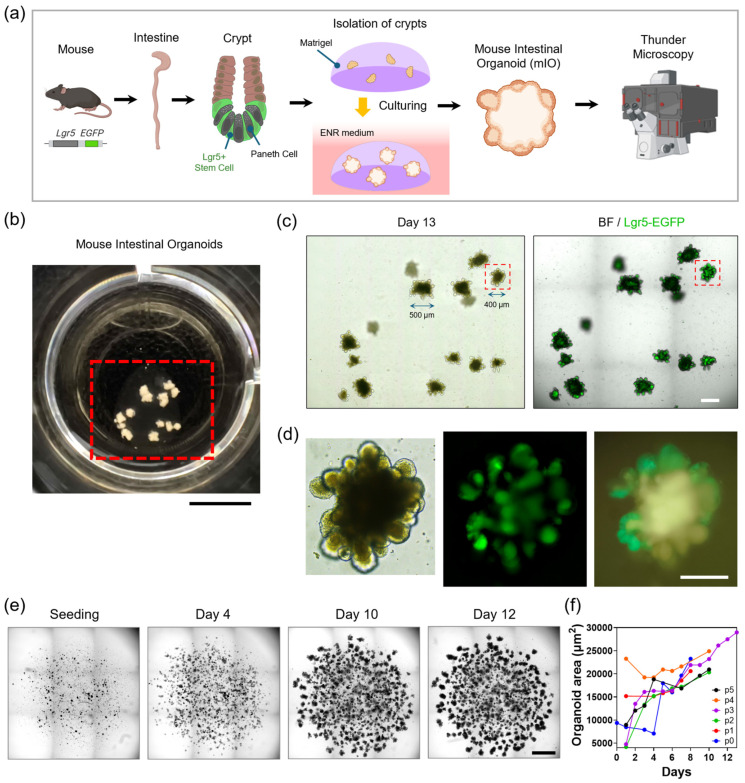
Long-term culture of mIOs derived from crypts of *Lgr5-EGFP-IRES-creERT2* mice. (**a**) Schematic of crypt isolation, Matrigel dome seeding, and culture workflow. (**b**) Photograph of mIOs cultured in a 24-well plate. Scale bar, 5 mm. (**c**) The red dashed box in (**b**) is observed by microscopy with 5× objective lens. Scale bar, 400 μm. (**d**) The red dashed box in (**c**) is magnified with 20× objective lens. Scale bar, 200 μm. (**e**) Large-area monitoring of organoid culture. Scale bar, 1 mm. (**f**) Variation in organoid area across passages.

To investigate the effect of DSS treatment on intestinal stem cells in organoids, we isolated intestinal crypts from *Lgr5-EGFP-IRES-creERT2* mice (Figure 2a). The crypts were embedded in Matrigel and maintained in complete ENR medium (EGF, Noggin, and R-spondin). The seeding density was 10^6^ cells/cm^3^ with a Matrigel dome volume of 50 μL, resulting in 50,000 cells per dome in 24-well plates. The growth of mIOs was monitored over 10–15 days of culture (Figure 2b). Color, bright-field (BF), and fluorescence images were obtained using a 5× objective lens (Figure 2c). The mIO sizes ranged from 400 to 500 μm. Individual mIOs were monitored using a 20× objective lens, which revealed EGFP fluorescence at the budding regions (Figure 2d). We confirmed that Lgr5-EGFP^+^ (green) stem cells were flanked by Paneth cells, consistent with the literature [30,31].

For large-area imaging, we employed an automated stage on an inverted microscope. Each Matrigel dome, covering approximately a 2 mm × 2 mm mosaic area, was monitored with a 5× objective lens (Figure 2e). Daily monitoring using this workflow captured variations in organoid distribution and morphology over time. Organoid areas were measured through daily observations for each passage (Figure 2f) [38].

### 3.3. Dextran Sulfate Sodium (DSS) Treatment to Induce Inflammation in Organoids

**Figure 3:**
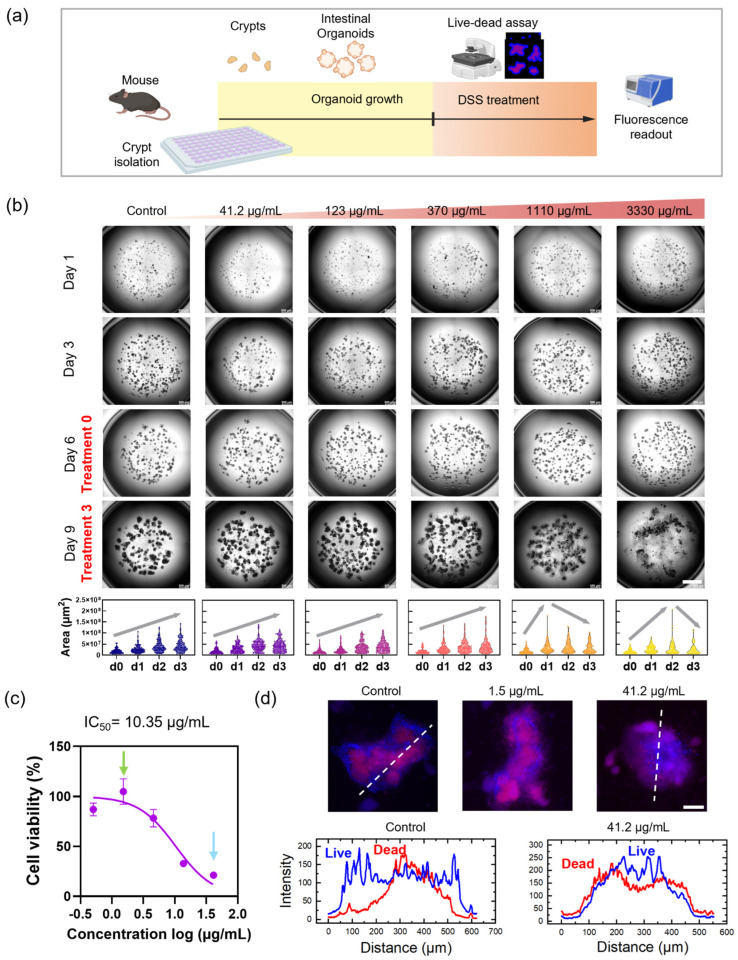
DSS treatment in mIOs. (**a**) Schematic of drug treatment experiment. (**b**) Longitudinal analysis showing DSS dose titration and daily microscopy observation. Scale bar, 1 mm. (**c**) Live–dead assay results. Green and blue arrows indicate 1.5 μg/mL and 41.2 μg/mL. (**d**) Fluorescence images of mIOs in live–dead assay. Scale bar, 100 μm. Fluorescence intensity profiles of Hoechst 33342 and propidium iodide for untreated and DSS-treated mIOs.

To explore CC imaging in conjunction with drug testing, we established a schematic workflow in which mIOs were treated with DSS to induce inflammation (Figure 3a). DSS is widely used to induce inflammatory bowel disease (IBD) models in mice both in vitro and in vivo [39,40,41]. We serially diluted DSS from 10,000 μg/mL by 1/3, resulting in concentrations ranging from 3330 μg/mL to 0.5 μg/mL (Supplementary Figure S2a). Daily monitoring of DSS-treated mIOs enabled longitudinal analysis (Figure 3b). Bright-field (BF) images of entire Matrigel domes were acquired using a 5× objective. For untreated mIOs, BF images at d9t3 (day 9, treatment day 3) showed typical growth patterns. With increasing DSS concentrations, epithelial disruption became progressively apparent. Large-area imaging of whole Matrigel dome regions revealed dose-dependent morphological changes across DSS titrations. We compared two different algorithms—AIVIA and OrganoSeg—which showed a similar tendency in the variation in organoid areas (Supplementary Figure S2b).

To assess mIO viability, we performed live–dead assays by treating organoids with Hoechst 33342 (live nuclear stain) and propidium iodide (dead cell marker), followed by measuring fluorescence readouts using a microplate reader (Molecular Devices iD3) (Figure 3c). Viability decreased with increasing DSS concentration, with an estimated IC_50_ of 10.35 μg/mL. The ratio of Hoechst 33342 to propidium iodide fluorescence correlated with fluorescence microscopy images (Figure 3d and Supplementary Figure S3). Since intestinal turnover is rapid (2–3 days), apoptotic cells accumulated inside the mIO lumen. In control organoids, dead cells remained within the lumen, whereas DSS-treated organoids exhibited dead cells spilling outside the epithelial layer, resembling a leaky gut model in which luminal contents escape from the mIOs.

### 3.4. Gene Expression and Transcriptomic Analysis of Inflammatory mIOs

**Figure 4:**
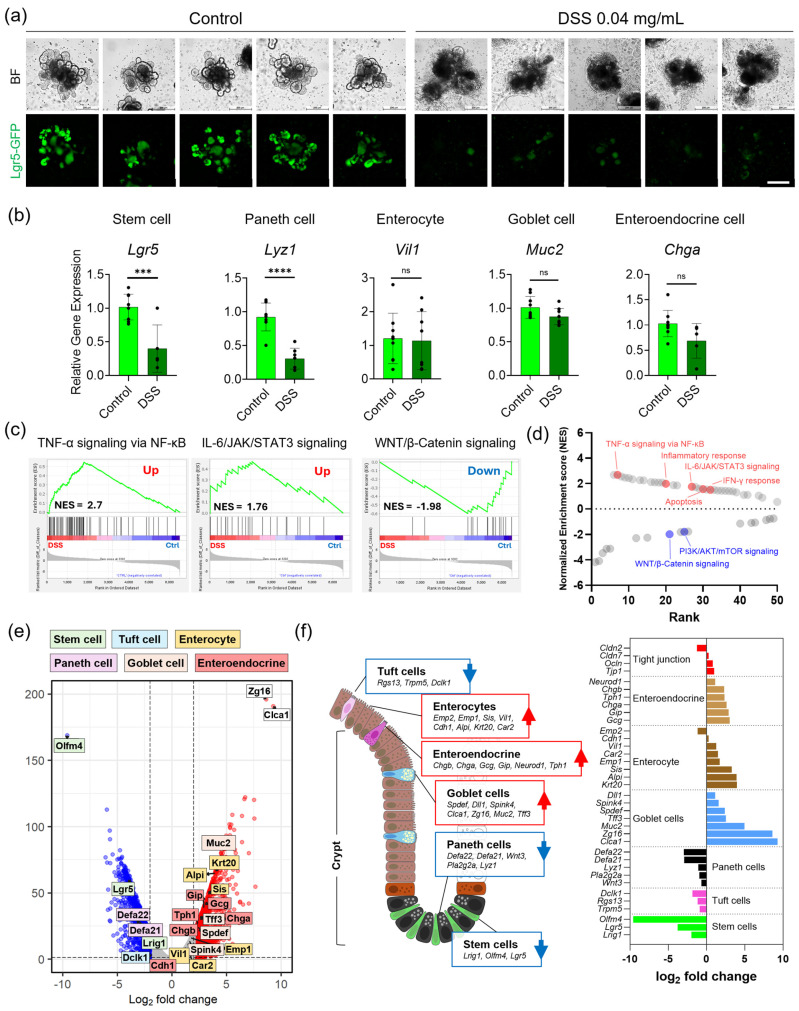
Gene expression by RT-PCR and mRNA sequencing for DSS-induced injury model of mIOs. (**a**) Fluorescence microscopy images of EGFP from Lgr5+ stem cells in control and DSS-treated mIOs. Scale bar, 200 μm. (**b**) Gene expression of intestinal markers *Lgr5*, *Lyz*, *Vil1*, *Muc2*, and *Chga*. Data represent the mean ± SD. **** *p* < 0.0001, *** *p* < 0.001, by unpaired two-tailed *t*-test. (**c**) Gene set enrichment analysis (GSEA) with normalized enrichment scores (NESs) for WNT/β-catenin (downregulation), IL-6/JAK/STAT3 (upregulation), and inflammatory signaling pathways (upregulation). (**d**) NES ranking plot for upregulated and downregulated pathways. (**e**) Volcano plot of downregulated and upregulated genes in DSS-treated mIOs. *Olfm4* was significantly downregulated. (**f**) DSS-induced injury model of mIOs.

Lgr5+ stem cells are the source of intestinal epithelial renewal [42,43]. In vivo DSS treatment is known to impair Lgr5+ intestinal stem cells [44]. To assess DSS-induced injury to Lgr5+ stem cells in our organoid model, we monitored Lgr5-EGFP fluorescence in control and DSS-treated mIOs. Microscopic observation using a 20× objective lens revealed markedly reduced EGFP fluorescence in DSS-treated mIOs (Figure 4a). Fluorescence intensity analysis from raw image data confirmed the diminished EGFP signal (Supplementary Figure S4), indicating that DSS treatment caused severe impairment of Lgr5+ stem cells due to direct epithelial injury.

Relative gene expression of intestinal cell type markers, including *Lgr5* (stem cell), *Lyz* (Paneth cell), *Vil1* (enterocyte), *Muc2* (goblet cell), and *Chga* (enteroendocrine cell), was measured by RT-PCR in DSS-treated mIOs (Figure 4b). Gene expression levels of *Lgr5* and *Lyz* were significantly decreased, while *Vil1*, *Muc2*, and *Chga* remained at similar levels, indicating preferential damage to the stem cell niche. These findings are consistent with previous reports that DSS induces direct epithelial injury and inflammation with loss of Lgr5+ stem cells, whereas TNBS or oxazolone induces immune responses with persistence of Lgr5+ stem cells [29].

To profile transcriptional changes, we performed mRNA-seq on DSS-treated mIOs versus controls and conducted gene set enrichment analysis (GSEA) with 50 Hallmark gene sets from the Molecular Signatures Database (MSigDB) (Figure 4c and Supplementary Figures S5 and S6) [45]. Enrichment analysis revealed upregulation of inflammatory pathways, including TNF-α signaling via NF-κB, IL-6/JAK/STAT3 signaling, inflammatory response, apoptosis, IFN-γ response, and p53 pathway, alongside the downregulation of stem cell maintenance and proliferation pathways, including WNT/β-catenin signaling and PI3K/AKT/mTOR signaling (Figure 4d).

In the volcano plot, Olfactomedin-4 (*Olfm4*), a crypt and intestinal stem cell-associated WNT-responsive marker [46], was significantly downregulated in DSS-treated mIOs (Figure 4e). Together with reduced WNT pathway activity, this decrease is consistent with impairment of the Lgr5+ stem cell population under DSS-induced direct epithelial injury. The reduced expression of *Olfm4* reflects injury to the stem cell niche and loss of Lgr5+ stem cells, with epithelial injury subsequently inducing inflammation as a secondary response [47,48]. We propose a DSS-induced injury model in which WNT/β-catenin signaling is downregulated with concomitant loss of Lgr5+ stem cells (Figure 4f) [49].

### 3.5. Image Segmentation of Intact and Injured Organoids

Image segmentation is widely employed for analyzing medical images, including MRI and CT scans [50,51]. In organoid research, this technique has evolved to characterize morphological image metrics such as area, perimeter, and circularity, facilitating applications in artificial intelligence and machine learning [16,35,37,52,53].

We applied the AIVIA image segmentation algorithm to analyze organoid structure. By using bright-field (BF) images [54,55], we tested organoid growth in both wild-type (WT, without gene modification) and Lgr5-EGFP models (Supplementary Figure S7), in which organoid area, perimeter, and circularity were comparable between the two models [30,31].

**Figure 5:**
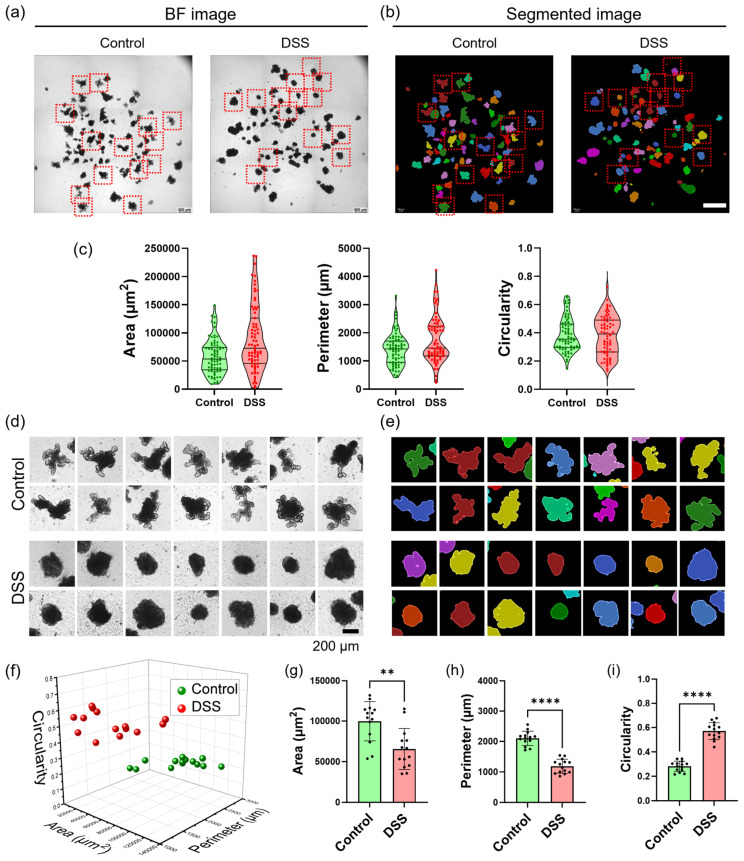
Image segmentation of intact and DSS-treated mIOs. (**a**) Microscopy images of mIOs under intact and DSS-treated conditions. Scale bar, 1 mm. (**b**) Segmented images. (**c**) Violin plots of area, perimeter, and circularity. (**d**) Selected mIOs images for the red dashed boxes in (**a**) under intact and DSS-treated conditions. Scale bar, 200 μm. (**e**) Selected segmented images for the red dashed boxes in (**b**). (**f**) 3D scatter plot of area–circularity–perimeter. (**g**–**i**) Comparison of morphological image metrics: area, perimeter, and circularity. Data represent the mean ± SD. **** *p* < 0.0001, ** *p* < 0.01 by unpaired two-tailed *t*-test.

We extended the image segmentation method to compare 80 intact and 86 DSS-treated organoids. BF and corresponding segmented images are shown (Figure 5a,b), along with violin plots of area, perimeter, and circularity (Figure 5c). Fourteen representative mIOs from each condition were analyzed to distinguish between intact and injured organoids (Figure 5d). BF images were segmented, with each organoid outlined and mesh-generated from the original microscopy images. Objects were segmented based on the mesh channel (Figure 5e). Non-treated intact mIOs exhibited well-developed budding geometries, whereas DSS-treated inflammatory mIOs showed epithelial disruption and collapsed morphology.

Extracted morphological image metrics were visualized by plotting area, perimeter, and circularity in a 3D graph (Figure 5f). Area, perimeter, and circularity were then compared between intact and DSS-treated mIOs (Figure 5g–i). Organoid area remained similar between conditions (Figure 5g). However, perimeter and circularity differed significantly. Intact mIOs displayed longer perimeters due to budding geometry compared to epithelium-disrupted mIOs (Figure 5h). DSS treatment induced organoid collapse, resulting in more rounded geometries and increased circularity (Figure 5i). Overall, the area and perimeter also tended to slightly decrease, due to the loss of cells and the degradation of budding geometry, respectively, while the circularity tended to increase due to the collapse of organoid geometry.

In addition, we analyzed an inflammatory model of human intestinal organoids (hIOs) derived from induced pluripotent stem cells (iPSCs) (Supplementary Figure S8) [12]. To induce inflammation in hIOs, we treated the organoids with proinflammatory cytokines such as TNF-α/IFN-γ. For this human inflammatory organoid model, we performed image segmentation analysis, through which we confirmed that the epithelial thickness decreased from 67.4 μm (control) to 34.5 μm (inflammatory model).

### 3.6. Image Tracing as CC-WF Vector to Estimate Integrity of mIOs

**Figure 6:**
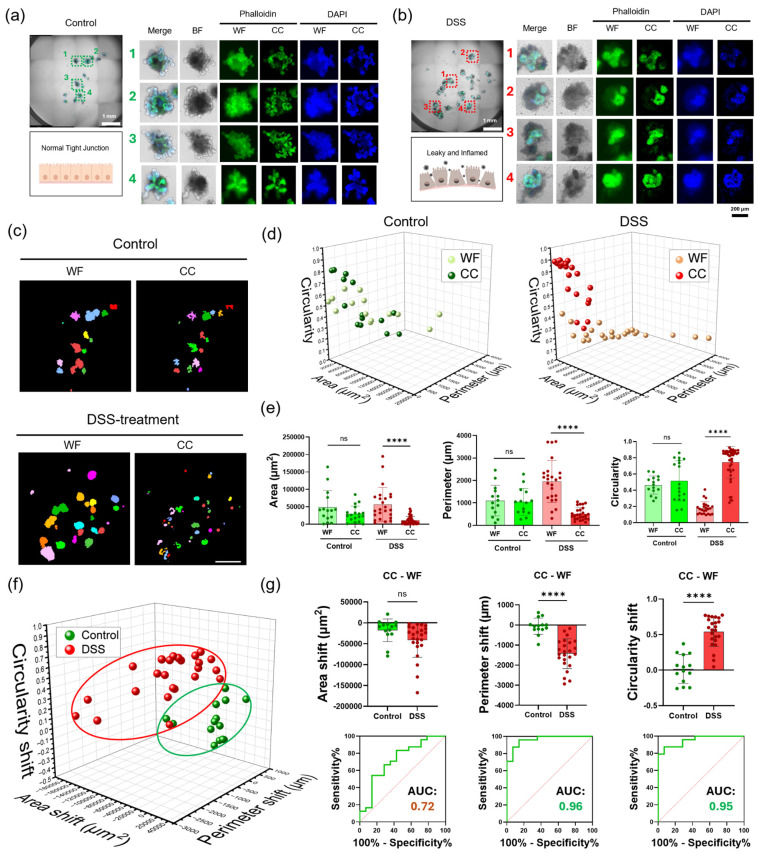
Image tracing analysis of healthy and DSS-treated (inflammatory) mIOs by CC-WF vectors. (**a**,**b**) WF fluorescence images of phalloidin- and DAPI-stained mIOs in healthy and DSS-treated states. (**c**) CC-processed fluorescence images. (**d**) Area–perimeter–circularity scatter plots of segmented objects from WF and CC for healthy and DSS-treated mIOs. (**e**) Area, perimeter, and circularity comparisons between WF and CC for healthy and DSS-treated mIOs. (**f**) CC-WF vector representation in area–perimeter–circularity 3D plot. (**g**) ROC curve analysis comparing healthy versus DSS-treated mIOs using CC-WF vector magnitude. Data represent the mean ± SD. **** *p* < 0.0001 by unpaired two-tailed *t*-test.

We explored image tracing as a CC-WF vector using DSS-treated mIOs (inflammatory intestinal organoids). For this analysis, mIOs were stained with phalloidin and DAPI (Figure 6). Intact mIOs exhibited sharp phalloidin green-fluorescence contrast along the lumen (Figure 6a), indicating F-actin integrity. In contrast, DSS-treated mIOs displayed a disrupted luminal structure with weak and diffuse phalloidin signal (Figure 6b).

To establish the image tracing workflow, we performed CC image processing on phalloidin-stained green fluorescence images from both intact and DSS-treated mIOs (Figure 6c). CC image processing substantially reduced background noise compared to WF images. For morphological parameter analysis, both CC and WF images underwent image segmentation. During segmentation, the target green fluorescence signal was converted to mesh representations, from which image metrics were extracted. Image metrics, including area, perimeter, and circularity, were quantified from these segmented objects. Area, perimeter, and circularity values were plotted in 3D scatter plots for intact and DSS-treated mIOs (Figure 6d). Using these morphological image metrics, we performed area, perimeter, and circularity analyses comparing WF and CC values between healthy and injured mIOs (Figure 6e) [56].

To explore image tracing, we calculated vector components representing the shift from WF to CC for healthy and injured mIOs (Figure 6f and Supplementary Figure S9) [57,58]. For healthy mIOs, CC-WF vectors showed minimal displacement, clustering near the origin (0, 0). In contrast, injured mIOs exhibited CC-WF vectors with directional displacement and substantially larger magnitude. The ROC curve analysis of CC-WF vector magnitudes of area, perimeter, and circularity is as follows (Figure 6g): the area shift shows an AUC of 72.3% (95% CI: 54.9–89.8%) with 66.7% sensitivity and a 28.6% false positive rate (1-specificity), the perimeter shift shows an AUC of 96.1% (95% CI: 90.7–100%) with 91.7% sensitivity and a 14.3% false positive rate (1-specificity), and the circularity shift shows an AUC of 95.2% (95% CI: 89.3–100%) with 87.5% sensitivity and a 7.1% false positive rate (1-specificity).

In summary, the perimeter shift and the circularity shift both yielded AUC = 0.95, whereas the area shift yielded AUC = 0.72, indicating that the area shift was less discriminative than the perimeter and circularity shifts. Therefore, integrating all three morphometrics provides a more reliable basis for discriminating control from DSS-treated organoids than relying on any single parameter alone. These results demonstrate that CC-WF vector-based image tracing can effectively distinguish mIO integrity states.

## 4. Discussion

One might question whether fluorescence intensity alone could serve as diagnostic criteria for organoid disease states, eliminating the need to compare CC and WF images. However, this overlooks a fundamental limitation of fluorescence microscopy: the discrepancy between what observers see through eyepieces and what sensitive CCD or CMOS cameras capture. High-sensitivity cameras can detect weak, non-specific fluorescence and display it using false colors (red, green, blue, magenta), potentially masking artifacts as apparent biological signals. Users must therefore verify whether captured signals originate from specific targets. Our image tracing approach—subtracting WF from CC images—directly addresses this challenge by reducing artifacts from non-specific fluorescence during digital image processing.

The second question concerns what changes occur during image tracing from WF to CC. Our procedure relies on image segmentation to extract pixel-based objects, from which quantitative metrics (area, circularity, perimeter) are calculated for both WF and CC images. Importantly, our image tracing workflow does not involve direct pixel-by-pixel image comparison but rather comparison of image-segmented objects from individual organoids [35,37]. While this segmentation-based approach may be considered a limitation for certain diagnostic applications, it offers a distinct advantage: the extracted metrics (area, perimeter, circularity) enable quantitative shape characterization of organoids using parameters familiar to conventional biomedical researchers rather than requiring specialized image-processing expertise.

Furthermore, the key diagnostic insight is that the magnitude of change between WF and CC—represented as a vector in area–perimeter–circularity 3D space—can systematically distinguish between intact and injured organoids. Injured organoids exhibit larger WF-to-CC shifts than intact organoids, providing the quantitative basis for diagnostic discrimination with high accuracy (AUC = 0.95).

Recently, machine learning (ML) and deep learning (DL) approaches have been applied to a range of biomedical fields, including the analysis of multi-layer omics data [59], classification of immune cells [60], and detection of labeled biomarkers [61]. The U-Net architecture has also become widely adopted in medical imaging in recent years [62,63,64]. Notably, SegFormer, a generalized image segmentation algorithm for noise reduction, has been implemented in microscopy imaging, demonstrating outstanding performance in tracking organoid growth even under severely noisy conditions [65]. Our approach described here can potentially be improved alongside such algorithmic advances. In particular, the budding structure of intestinal organoids and its collapse upon DSS treatment and subsequent inflammation require even more precise segmentation procedures, capable of distinguishing budding features from background debris-induced noise and out-of-focus hazed regions.

## 5. Conclusions

We demonstrated that computational clearing serves not only as an image enhancement method but also as a diagnostic tool for quantitative phenotyping of intestinal organoids. By introducing the differential vector CC-WF, we extended the imaging-derived differences into quantitative measures of epithelial integrity, enabling objective assessment of DSS-induced injury in mIOs. DSS treatment induced characteristic imaging phenotypes, including luminally accumulated dead cells redistributed outside of mIOs, that correlated with transcriptomic signatures of inflammatory activation (IL-6/JAK/STAT3 and TNF-α/NF-κB upregulation) and suppression of stem-cell/homeostatic pathways (WNT/β-catenin and PI3K/AKT/mTOR downregulation). The CC-WF image tracing workflow enabled high-throughput monitoring across large organoid populations and integrated effectively with image segmentation algorithms. Together, these findings position CC-WF-based image tracing as a practical bridge connecting microscopy platforms to quantitative diagnostic applications in organoid-based disease modeling and drug screening.

## Data Availability

The image data presented in this study are available in the Zenodo repository, https://zenodo.org/records/18627307 (Version v1, accessed on 13 February 2026). Gene expression profiles are available in the NCBI Gene Expression Omnibus under accession number GSE328646, https://www.ncbi.nlm.nih.gov/geo/query/acc.cgi?acc=GSE328646 (accessed on 26 April 2026).

## Supplementary Materials

The following supporting information can be downloaded at: https://www.mdpi.com/article/10.3390/nano16100629/s1, Figure S1. Comparison of Widefield (WF), Computational clearing (CC) and Confocal fluorescence images. Figure S2. Dextran sulfate sodium (DSS) concentrations and image-based analysis of DSS-induced organoids using segmentation. Figure S3. IC50 analysis and fluorescence imaging of live-dead assays. Figure S4. Fluorescence intensity versus pixel for Lgr5-EGFP mouse intestinal organoids (mIOs). Figure S5. Upregulated gene sets from gene set enrichment analysis (GSEA) of DSS-treated mIOs. Figure S6. Downregulated gene sets from GSEA of DSS-treated mIOs. Figure S7. Wild type and Lgr5-EGFP mIOs exhibit comparable morphological properties. Figure S8. Histological characterization of human intestinal organoids (hIOs) via H&E staining. Figure S9. Normalized distance measurement of CC-WF for ROC curve analysis. Figure S10. Image segmentation workflow by AIVIA. Table S1. Primer list.

## Abbreviations

The following abbreviations are used in this manuscript:

CC	Computational Clearing
WF	Widefield
mIO	Mouse Intestinal Organoid
ENR	Epidermal Growth Factor, Noggin, and R-spondin
DSS	Dextran Sulfate Sodium
BF	Bright-Field
IBD	Inflammatory Bowel Disease
EGFP	Enhanced Green Fluorescent Protein
GSEA	Gene Set Enrichment Analysis
NES	Normalized Enrichment Score
TNF	Tumor Necrosis Factor
NF-κB	Nuclear Factor Kappa-light-chain-enhancer of Activated B Cells
IL	Interleukin
JAK	Janus Kinase
STAT	Signal Transducer and Activator of Transcription
IFN	Interferon
WNT	Wingless and Int-1
PI3K	Phosphoinositide 3-Kinase
AKT	AKT Serine/Threonine Kinase (Protein Kinase B)
mTOR	Mechanistic Target of Rapamycin
Olfm4	Olfactomedin 4
MRI	Magnetic Resonance Imaging
CT	Computed Tomography
DAPI	4′,6-Diamidino-2-Phenylindole
ROC	Receiver Operating Characteristic
AUC	Area Under the Curve
hIO	Human Intestinal Organoid
