# Supplementary material for: Image Tracing of Inflammatory Intestinal Organoids via Computational Clearing
Source: Nanomaterials (Basel). 2026 May 19;16(10):629. doi: 10.3390/nano16100629 (PMC13209662; doi:10.3390/nano16100629)
Supplement: Supplementary file 1 [file nanomaterials-16-00629-s001.zip › nanomaterials-4225391-supplementary.pdf]

Supplementary Information

Image Tracing of Inflammatory Intestinal Organoids via Computational Clearing

Dong-Gyu Jeon[1,2], Min-Young Han[3], Hana Lee[4], Hanguk Hwang[3], Ji-Min Lee[2], Eun Soo Kim[5], Gang Ho Lee[6], Yongmin Chang[1,3], Mi-Young Son[4], Mae-Ja Park[7], Sung-Wook Nam[1,2,3] *

Supplementary Figure S1. Comparison of Widefield (WF), Computational clearing (CC) and Confocal fluorescence images

Supplementary Figure S2. DSS concentrations and image-based analysis of DSS-Induced organoid using segmentation

Supplementary Figure S3. IC_{50} analysis and fluorescence imaging of live-dead assays

Supplementary Figure S4. Fluorescence intensity versus pixel for Lgr5-EGFP mouse intestinal organoids (mIOs)

Supplementary Figure S5. Upregulated gene sets from gene set enrichment analysis (GSEA) of dextran sulfate sodium (DSS)-treated mouse intestinal organoids (mIOs)

Supplementary Figure S6. Downregulated gene sets from GSEA of DSS-treated mIOs

Supplementary Figure S7. Wild type and Lgr5-EGFP mIOs exhibit comparable morphological properties

Supplementary Figure S8. Histological characterization of hIOs via H&E staining

Supplementary Figure S9. Normalized distance measurement of CC-WF for ROC curve analysis

Supplementary Figure S10. Image segmentation workflow by AIVIA

Supplementary Table S1. Primer list

Comparison of Widefield (WF), Computational clearing (CC) and Confocal fluorescence images

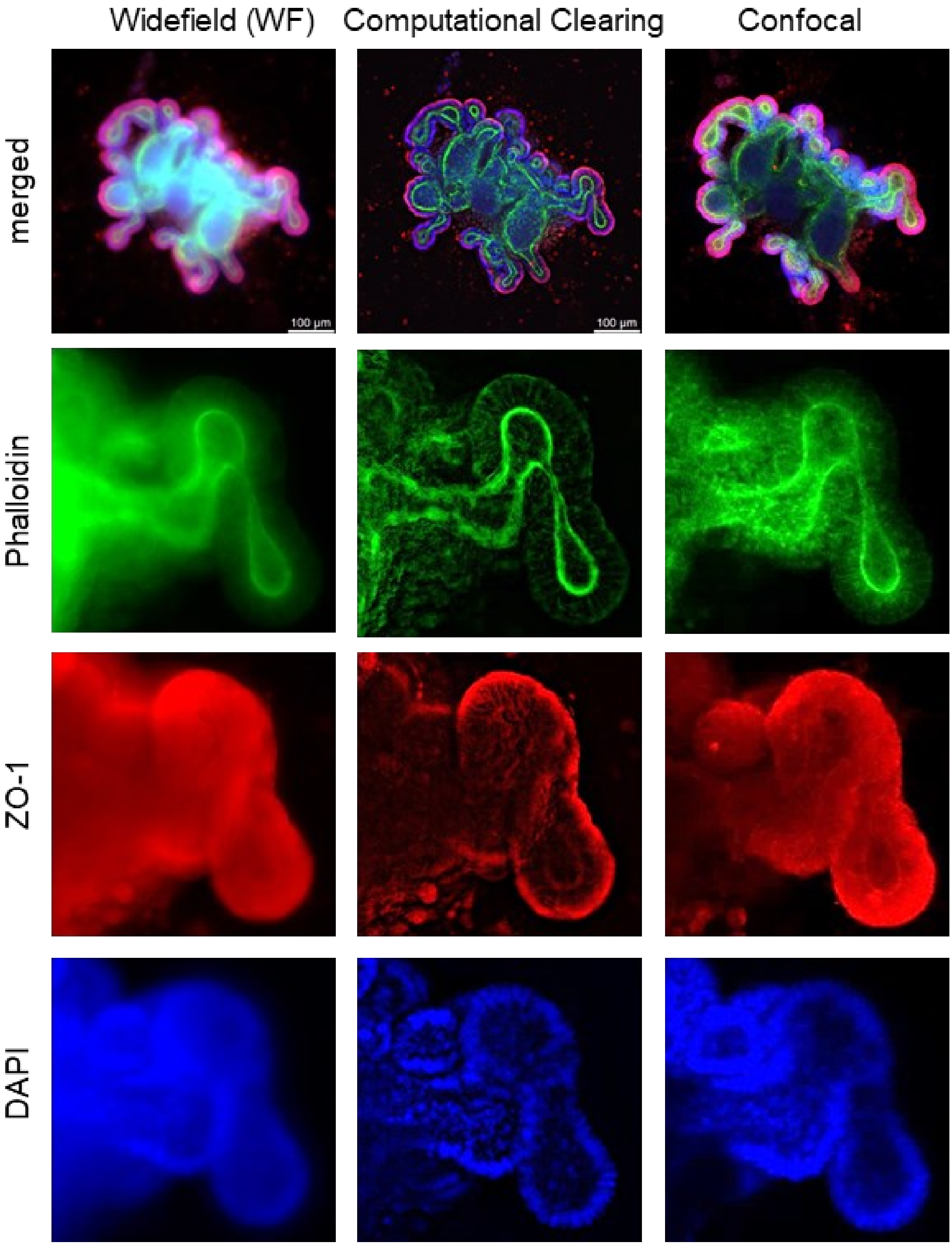

Supplementary Figure S1. Widefield (WF) images were processed into Computational Clearing (CC) images using LAS X software. The same organoid was analyzed by confocal microscopy. The CC process effectively removed background noise and improved image sharpness, yielding results comparable to the confocal images.

DSS concentrations and image-based analysis of DSS-induced organoids using segmentation

(a)

Dilution ratio of DSS from 10,000 μg/mL	1/3	1/9	1/27	1/81
Concentration	3330 μg/mL	1110 μg/mL	370 μg/mL	123 μg/mL
1/243	1/729	1/2187	1/6561	1/19683
41.2 μg/mL	13.7 μg/mL	4.6 μg/mL	1.5 μg/mL	0.5 μg/mL

(b)

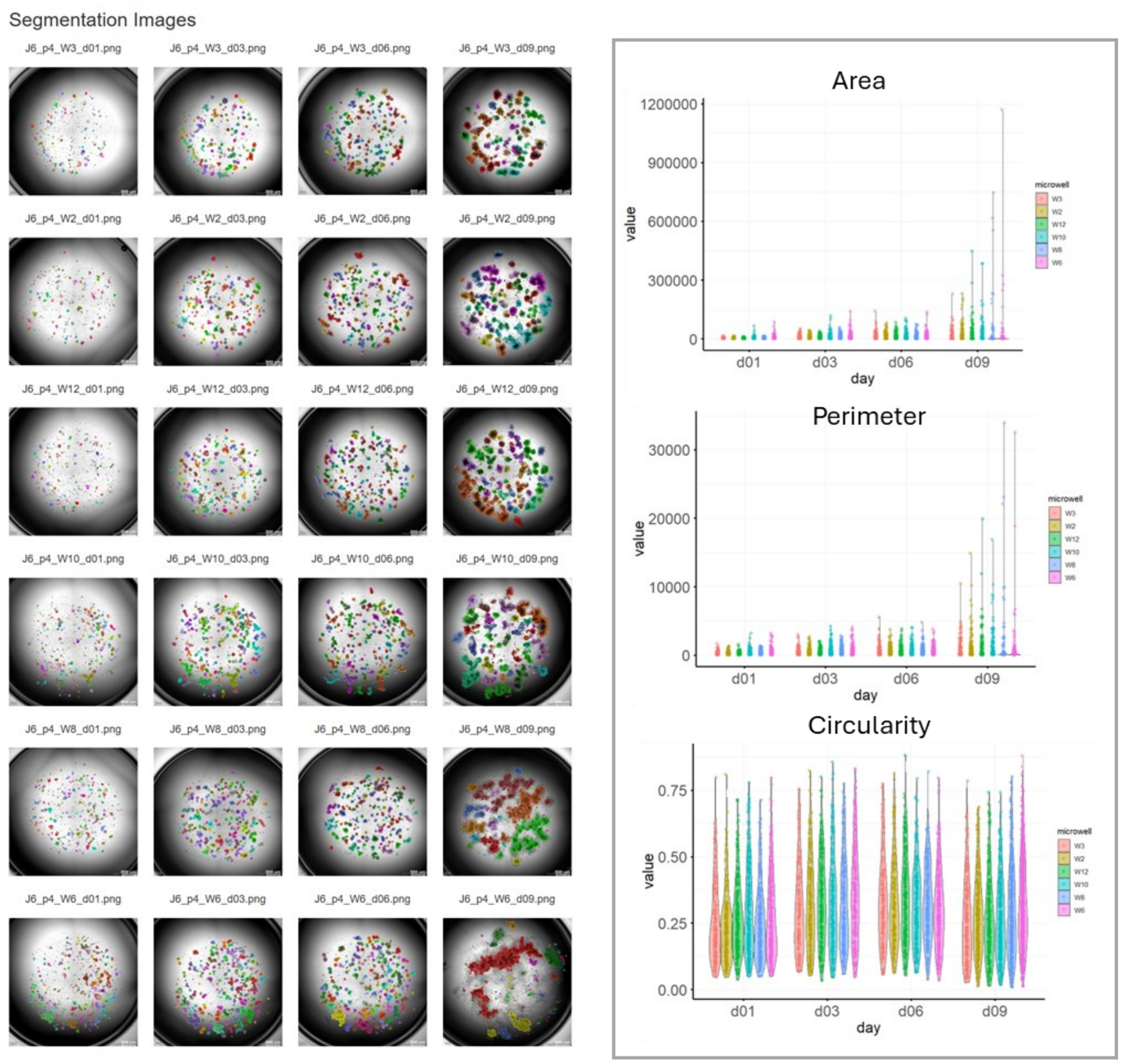

Supplementary Figure S2. (a) DSS concentrations were prepared via a 1/3 serial dilution starting from 10,000 μg/mL. (b) Daily images of organoids were segmented using AIVIA software. Morphometric analysis of organoids, including area, perimeter, and circularity plots.

IC_{50} analysis and fluorescence imaging of live-dead assays

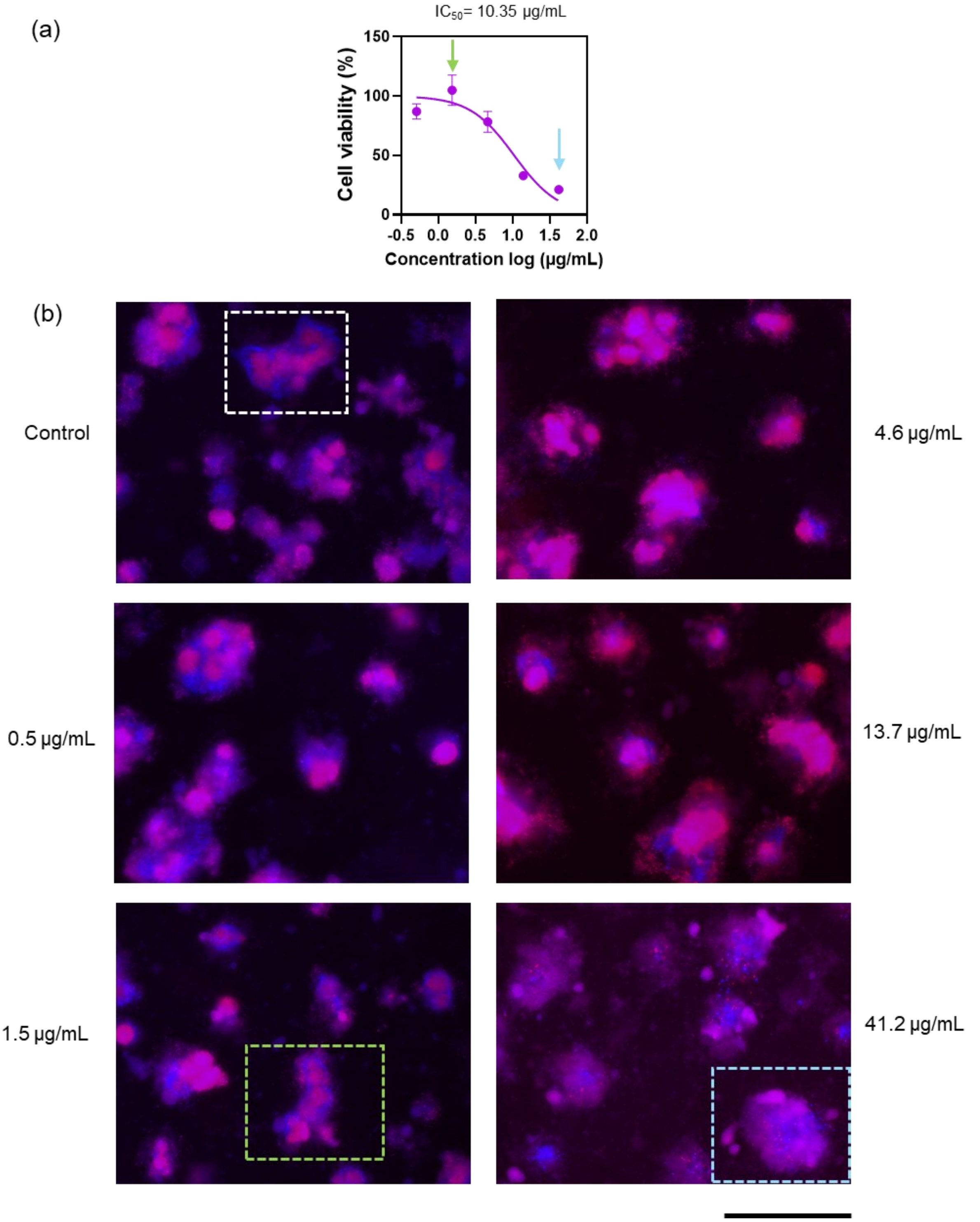

Supplementary Figure S3. (a) IC_{50} analysis derived from live-dead assay results. Green arrow indicates 1.5 μg/mL and blue arrow indicates 41.2 μg/mL (b) Representative fluorescence images of mIOs in the live-dead assay. The dotted rectangular areas correspond to the magnified images shown in Figure 3d. Scale bar, 500 μm.

Fluorescence intensity versus pixel for EGFP-Lgr5 mouse intestinal organoids (mIOs)

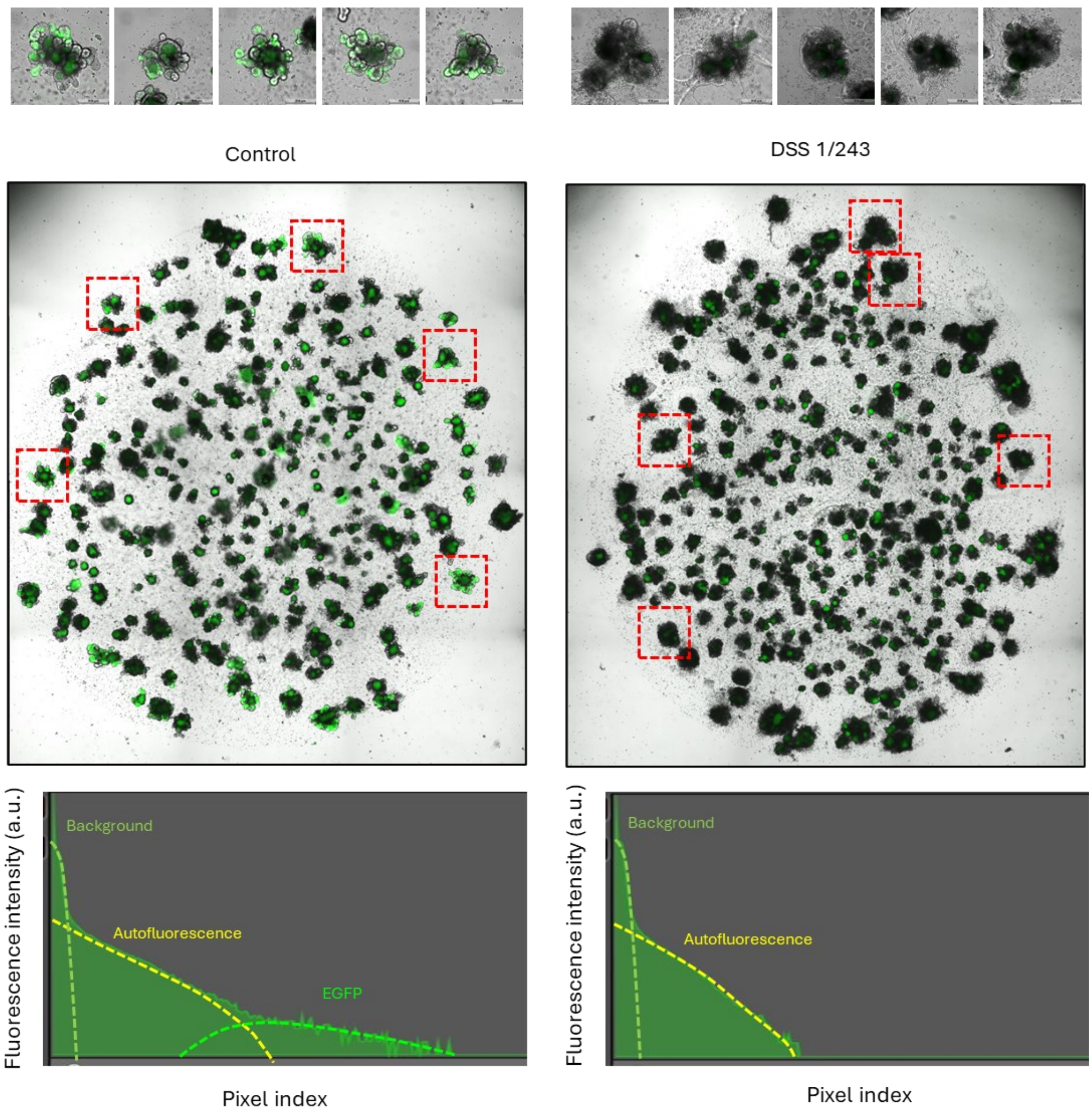

Supplementary Figure S4. Pixel intensity distribution of EGFP fluorescence in control and DSS-treated mIOs. Intensity values were extracted from raw 16-bit images using LAS X software. Control mIOs exhibit clear Lgr5-EGFP signals with high-intensity pixels, whereas DSS-treated mIOs lack Lgr5-EGFP signals.

Upregulated gene sets from gene set enrichment analysis (GSEA) of dextran sulfate sodium (DSS)-treated mouse intestinal organoids (mIOs)

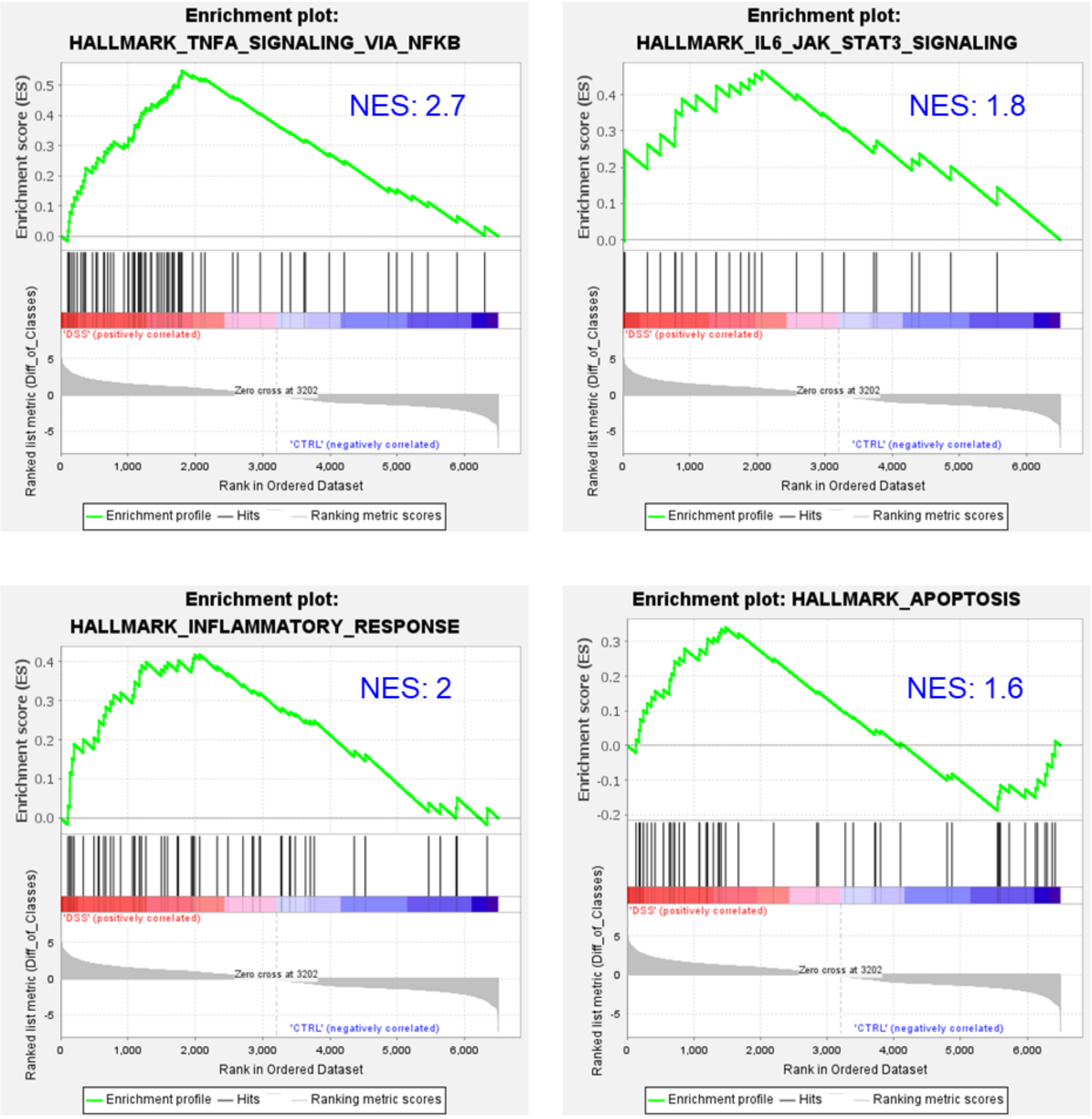

Supplementary Figure S5. Upregulated gene sets including TNF-α signaling via NF-κB, IL6-JAK-STAT3 signaling, inflammatory response, and apoptosis.

Downregulated gene sets from GSEA of DSS-treated mIOs

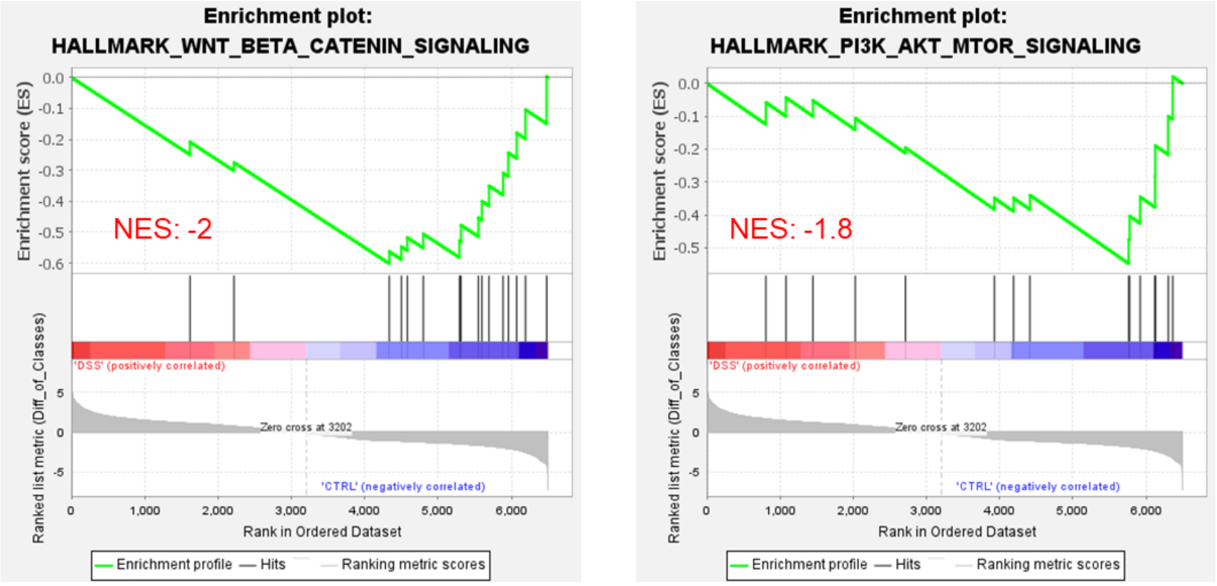

Supplementary Figure S6. Downregulated gene sets including WNT/β-catenin signaling and PI3K-AKT-mTOR signaling.

Wild type and Lgr5-EGFP mIOs exhibit comparable morphological properties

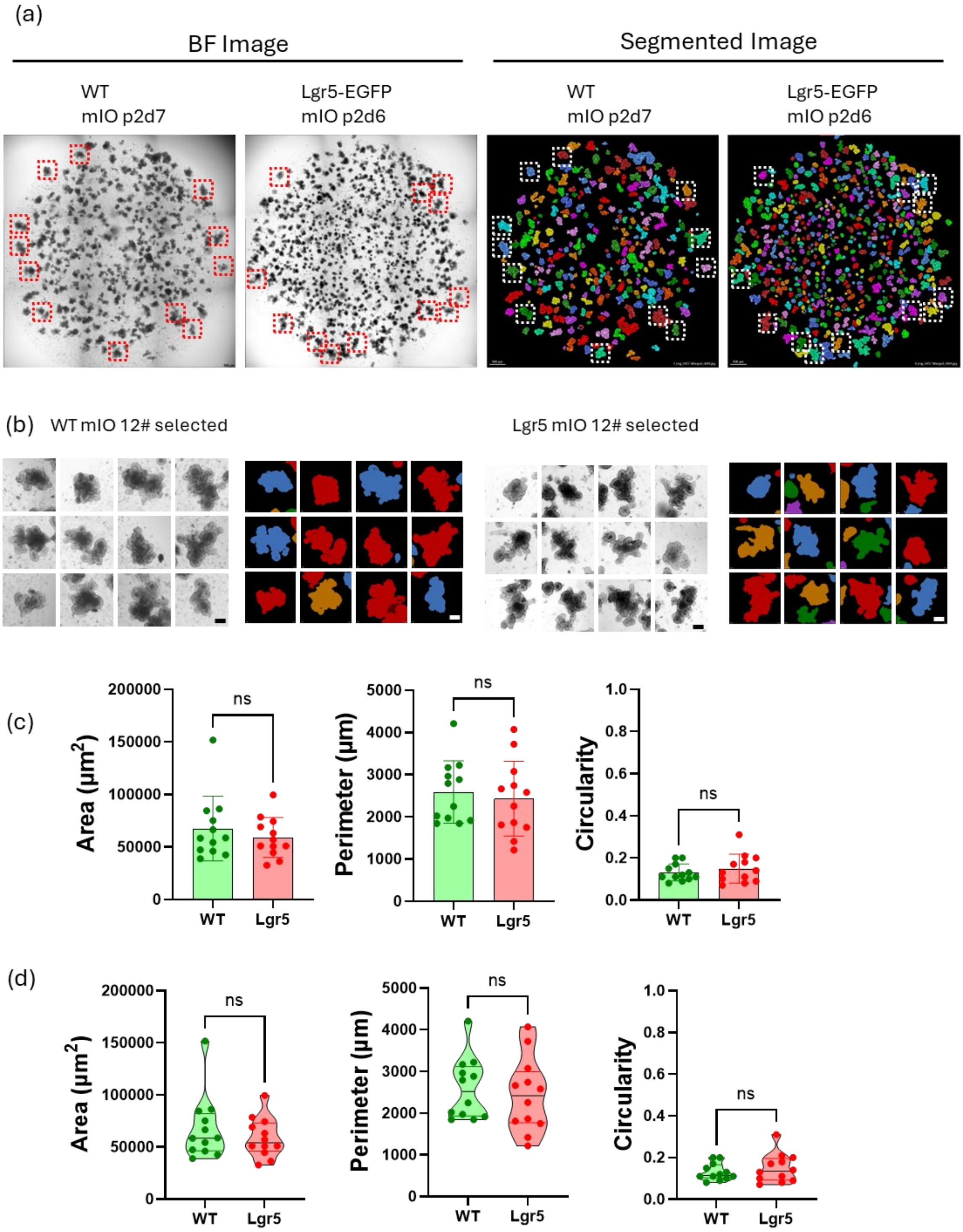

Supplementary Figure S7. (a) Bright-field (BF) images of WT mIOs and Lgr5-EGFP mIOs were segmented using AIVIA. (b) Twelve mIOs from each group were manually annotated. (c, d) Quantitative analysis of the segmented images, including area, perimeter, and circularity, revealed no significant differences between WT mIOs and Lgr5-EGFP mIOs.

Histological characterization of human intestinal organoids (hIOs) via H&E staining

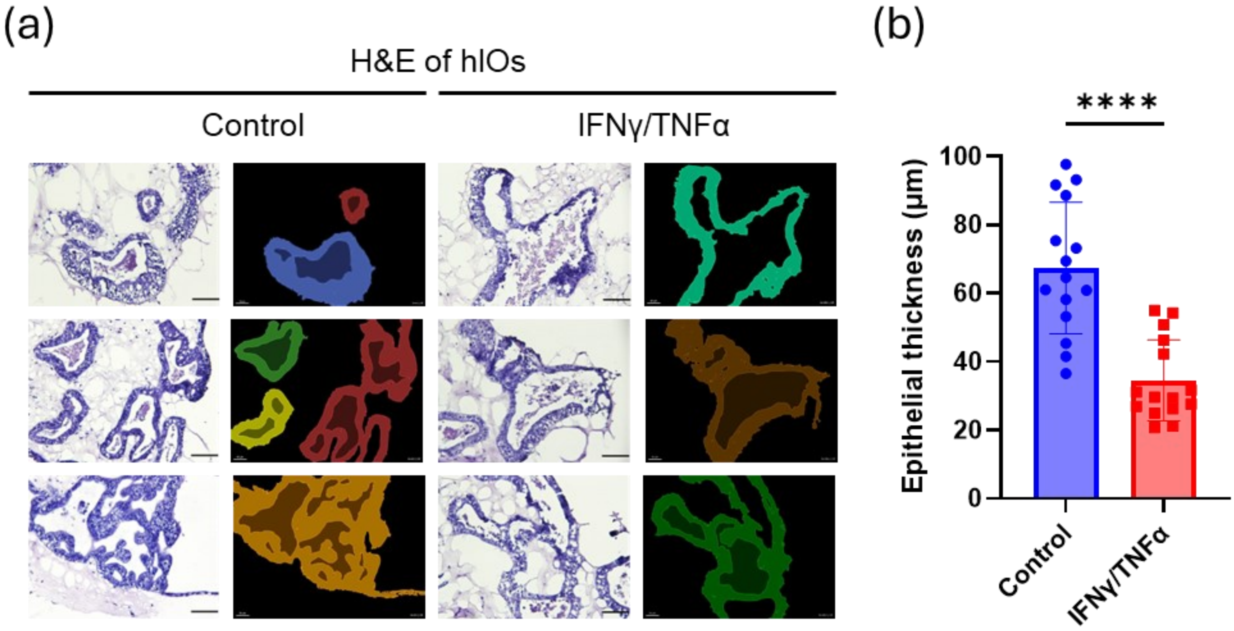

Supplementary Figure S8. To compare control and inflammatory conditions in hIOs, IFN-γ and TNF-α were used to induce inflammation. (a) H&E-stained images were segmented using AIVIA software. (b) The epithelial thickness was measured to be 67.4 μm in the control group and 34.5 μm in the inflammatory group.

Normalized distance measurement of CC-WF for ROC curve analysis

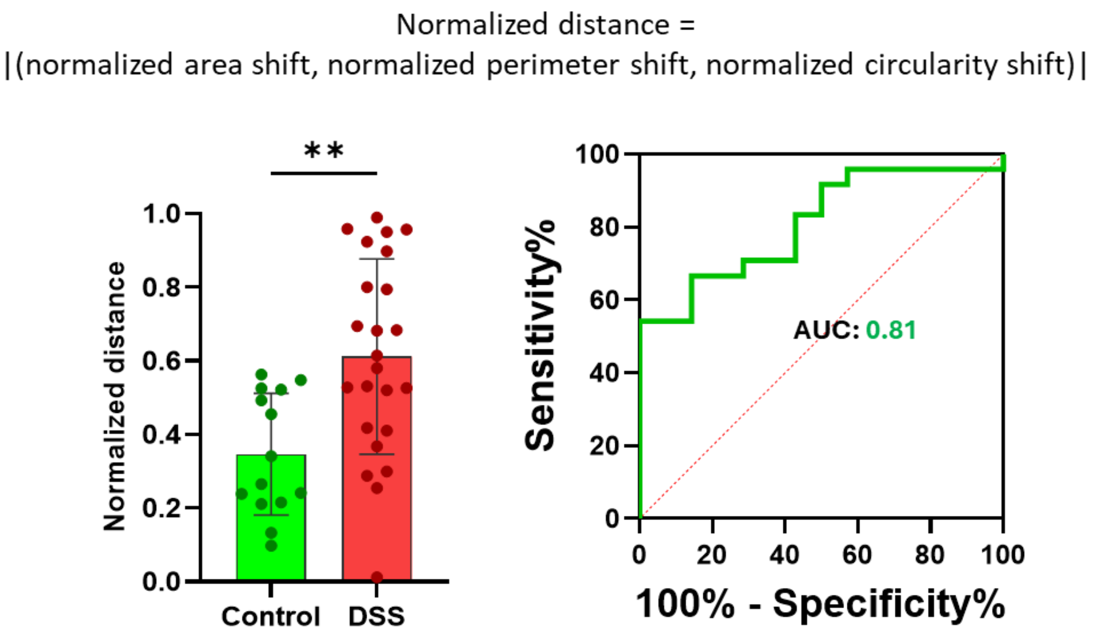

Supplementary Figure S9. For the shifted distance of CC-WF, a normalized distance calculation method was applied. The normalized distance was 0.35 in the control group and 0.61 in the injured group. ROC curve analysis yielded an AUC of 81% (95% CI: 67.33%-94.57%)

Image segmentation workflow by AIVIA

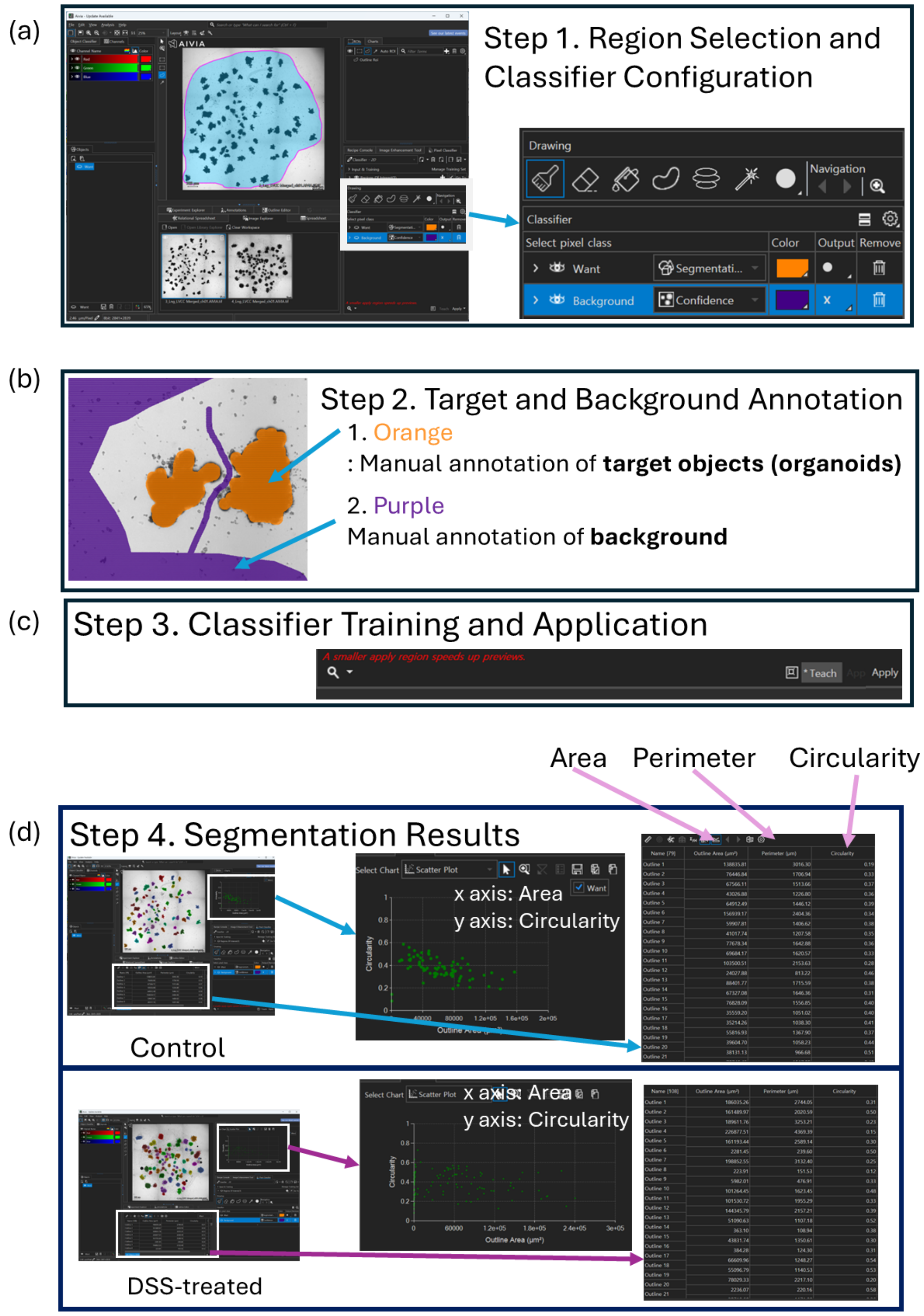

Supplementary Figure S10. AIVIA workflow (a) Selection of the region of interest (ROI) and switching of the classifier mode from confidence to segmentation for the target class. (b) Manual annotation of representative target objects and background regions, performed separately on selected areas. (c) Training of the classifier in AIVIA using the annotations in (b), followed by application of the trained model to the entire image. (d) Visualization of the full segmentation results, together with quantitative outputs including scatter plots and spreadsheets containing morphological measurements (area, perimeter, and circularity).

Primer list

Primer		Sequence
Lgr5	Fw	ACATTCCCAAGGGAGCGTTC
	Rev	ATGTGGTTGGCATCTAGGCG
Lyz	Fw	GAGACCGAAGCACCGACTATG
	Rev	CGGTTTTGACATTGTGTTCGC
Vil1	Fw	TCAAAGGCTCTCTCAACATCAC
	Rev	AGCAGTCACCATCGAAGAAGC
Muc2	Fw	ATGCCCACCTCCTCAAAGAC
	Rev	GTAGTTTCCGTTGGAACAGTGAA
Chga	Fw	CCAAGGTGATGAAGTGCGTC
	Rev	GGTGTCGCAGGATAGAGAGGA
Gapdh	Fw	TGGCCTTCCGTGTTCCTAC
	Rev	GAGTTGCTGTTGAAGTCGCA

Supplementary Table S1. Primer sequences of *Lgr5, Lyz, Vil1, Muc2, Chga,* and *Gapdh*
